# Alcohol or Stress Exposure During Late Adolescence Impairs Risk Assessment Later in Life, Disrupting the Ethological Structure of Behavior in Mice

**DOI:** 10.1111/acer.70328

**Published:** 2026-07-10

**Authors:** Beatriz Deo Sorigotto, Isabel Marian Hartmann de Quadros, Karina Possa Abrahao

**Affiliations:** ^1^ Programa de Pós‐Graduação em Psicobiologia, Escola Paulista de Medicina Universidade Federal de São Paulo São Paulo Brazil; ^2^ Departamento de Psicobiologia, Escola Paulista de Medicina Universidade Federal de São Paulo São Paulo Brazil; ^3^ Departamento de Fisiologia, Escola Paulista de Medicina Universidade Federal de São Paulo São Paulo Brazil

**Keywords:** C57BL/6, decision‐making, ethanol, ethoexperimental analysis, sex differences

## Abstract

**Background:**

Decision‐making relies on risk assessment as a critical process that enables organisms to evaluate threats before exploring potentially dangerous areas. While adolescence is a period of heightened vulnerability to external factors such as substance use and stress, it remains unclear how these factors disrupt risk assessment, particularly at the level of behavior structure: sequential actions, organization and transition probabilities between defensive and exploratory behaviors. We investigated whether repeated ethanol intoxication or pharmacological stress during late adolescence alters behavioral structure in early adulthood.

**Methods:**

Male and female C57BL/6 mice received four intermittent injections of saline, ethanol (4.0 g/kg), or the α2‐adrenergic antagonist yohimbine (2.0 mg/kg) during late adolescence (7–8^th^ weeks). In early adulthood (9^th^ week), animals were tested in the Light–Dark Box and Elevated Plus Maze. In addition to measuring classical anxiety‐like and exploratory behavior, we focused on risk assessment behaviors (stretches/head‐outs) categorized as NoGo (evaluation without entering the aversive area) or Go (evaluation followed by entry). We used 1^st^ order transition probability Markov chain analyses to map the transitions between these states, defining the behavioral structure.

**Results:**

Both ethanol and yohimbine produced persistent reductions in NoGo risk assessment behaviors in adulthood, in both sexes, with minimal effects on classical anxiety‐like and exploratory measures. Transition probability and correlation analyses revealed a reorganization of behavioral structure, characterized by reduced transitions toward risk assessment events and a bias toward risk‐taking actions.

**Conclusions:**

These findings indicate that repeated late adolescent alcohol intoxication or stress exposure induces long‐lasting alterations in risk assessment and decision‐making strategies.

## Graphical abstract

Alcohol and stress during adolescence may lead to long‐lasting impairments in risky decision‐making. We show that adolescent ethanol or yohimbine exposure selectively reduces cautious risk assessment without changing anxiety‐like behaviors, reorganizing behavioral structure. Late adolescence emerges as a critical window for durable reshaping of decision‐making under threat.

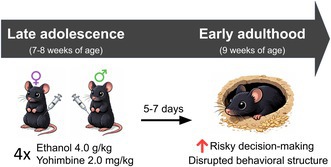

## 1 Introduction

A central aspect of decision‐making is risk assessment, a defensive behavioral process through which an organism, confronted with potential or ambiguous threats, detects and analyzes both the threat stimulus and the surrounding context (Blanchard et al. 1989, 2011). Risk assessment behaviors are considered to be closely associated with anxiety‐like behaviors (Carobrez and Bertoglio 2005) and to reflect a conflict between approach and avoidance tendencies (Blanchard et al. 1991). In rodents, such behaviors are commonly investigated using experimental paradigms that mimic instinctive approach‐avoidance conflict, like classical anxiety‐related tasks such as the Light–Dark Box (LDB) and the Elevated Plus Maze (EPM) (Bourin and Hascoët 2003; Kraeuter et al. 2018).

External factors such as drugs of abuse and stressors can interfere with approach and avoidance behaviors. For example, the anxiolytic effect of acute ethanol, which is measured by increased approach and decreased avoidance of aversive locations, has been extensively demonstrated in mice (Gulick and Gould 2009; LaBuda and Fuchs 2001) and rats (Acevedo et al. 2014; Bertoglio and Carobrez 2002) when tested in paradigms such as the EPM. Thereby, it is possible that alcohol can also disrupt risk assessment by shifting the balance between exploratory and avoidance states, leading to maladaptive changes in the ethological structure of behavior. Importantly, these effects are not restricted to acute ethanol intoxication. Chronic binge alcohol drinking followed by protracted abstinence induces persistent behavioral alterations, including reduced avoidance and risk assessment, as well as increased approach behavior, noted as increased compulsive‐like behavior in mice (Rivera‐Irizarry et al. 2023). Notably, ethanol exposure during adolescence leads to behavioral alterations that persist into adulthood, including changes in exploratory and emotional behaviors, as well as increased alcohol consumption (Amodeo et al. 2018).

In addition to its direct pharmacological effects, ethanol activates the hypothalamic–pituitary–adrenal (HPA) axis (Quadros et al. 2016), acting as a stressor. Likewise, stressful experiences are powerful modulators of risk assessment and decision‐making, although their behavioral consequences depend on the nature, intensity, and timing of the stressor. For example, predator odor stress increases anxiety‐like behaviors while reducing risk assessment and exploratory activity in rats in the EPM (Adamec et al. 2001), whereas social defeat stress increases risk assessment in rats exposed to the same test (Stickling and Rosenkranz 2025). Conversely, in humans, social stressors such as financial strain also modulate inhibitory control (Hughes et al. 2024), possibly modulating risk‐taking behaviors.

These findings suggest that alcohol intoxication and stress exposure can alter how animals evaluate and respond to potentially threatening environments, but they do not rule out the possibility of long‐term changes that emerge later in life. This is of particular interest when considering developmental stages, especially adolescence. Neural systems involved in decision‐making undergo protracted maturation throughout adolescence, rendering this period especially vulnerable to environmental perturbations (Spear 2013, 2018). In mice, this period spans approximately from 3 to 8 weeks of age (Bell 2018), with early adolescence (∼3–5 weeks) corresponding to rapid limbic and motivational circuit maturation, and late adolescence (∼6–8 weeks) aligning with continued development of prefrontal and corticostriatal systems implicated in cognitive control and decision‐making (Gutierrez‐Castellanos et al. 2024; Kasanetz and Manzoni 2009; Pöpplau et al. 2024; Tarazi et al. 1998). Notably, these stages are considered broadly analogous to early‐mid and late adolescence in humans (Dutta and Sengupta 2016; Semple et al. 2013).

Exposure to alcohol or stress during adolescence has been associated with long‐lasting alterations in affective and motivational behaviors that persist into adulthood. For example, studies in mice show that stress exposure during early and late adolescence (3–8 weeks) increases anxiety‐like behaviors later in life (9 weeks; Caruso et al. 2018), and ethanol exposure during early adolescence alters ethanol sensitivity and heightens anxiety‐like and risky behaviors in adult males (Pichinin et al. 2026). Less is known about how late adolescent alcohol or stress exposure reshapes the organization of decision‐making, particularly during risk assessment, when mice reach adulthood.

Beyond the quantification of individual behaviors, risk assessment can be better understood by integrating it into the decision to take or not take the risk and through the temporal organization and sequencing with other actions. In ethological and ethoexperimental contexts (Blanchard et al. 1989), the way behaviors are chained over time provides critical insight into underlying decision strategies, revealing patterns that are not captured by measures of frequency or duration alone (Brown and de Bivort 2018). Analytical approaches based on behavioral transitions, which can be represented by Markov chains (Sanabria et al. 2019; Tejada et al. 2010), offer a powerful framework to characterize probabilistic relationships between successive behaviors and to examine how animals navigate approach‐avoidance conflicts. By using Markov chain models, experimenters can analyze behavior as sequences of state transitions. These methods have been increasingly used to identify alterations in behavioral organization associated with anxiety, stress, and maladaptive decision‐making (Arantes et al. 2013; Maubourguet et al. 2008; Tejada et al. 2010).

In the present study, we investigated whether repeated heavy ethanol intoxication or pharmacological stress (administration of yohimbine, an α2‐adrenergic receptor antagonist that increases noradrenaline release and is widely used as a pharmacological stressor in relapse models to reinstate drug seeking; Chen et al. 2015; Shepard et al. 2004) during late adolescence produces persistent alterations in anxiety‐like, exploratory, and risk assessment behaviors in adulthood. Female and male mice were exposed to ethanol or yohimbine during late adolescence and later evaluated in the LDB and EPM, two paradigms that allow the characterization of approach‐avoidance conflict and risk assessment. In parallel, blood ethanol concentrations were measured to assess intoxication levels during the exposure period. By focusing on behavioral outcomes that persist beyond the acute exposure window, this study aims to clarify how adolescent alcohol and stress experiences bias adult decision‐making.

## 2 Materials and Methods

### 2.1 Animals

All procedures followed institutional guidelines aligned with national laws and regulations (Comissão de Ética no Uso de Animais; CEUA #7826300522; Universidade Federal de São Paulo, UNIFESP), and were performed in agreement with the NIH Guide for the Care and Use of Laboratory Animals (NIH 2011). We used adolescent C57BL/6 female and male mice (6 weeks old on arrival; *n* = 59). All mice were purchased from the Centro de Desenvolvimento de Modelos Experimentais para Medicina e Biologia (CEDEME/UNIFESP, Brazil). Animals were housed in groups of 3–4, separated by sex, in polypropylene cages (44 × 34 × 16 cm) placed in ventilated racks (Alesco, Monte Mor, SP, Brazil) under a 12 h light/dark cycle with lights on at 7:00 a.m. Chow (Nuvilab CR‐1, Nuvilab, Curitiba, PR, Brazil) and water were available *ad libitum* throughout the study. Prior to the beginning of experiments, animals were allowed to acclimate to the *vivarium* for 1 week and underwent handling and weighing by the experimenter. Card paper rolls were used for environmental enrichment, and cages were changed once a week.

### 2.2 In Vivo Drug Exposure Protocol

**FIGURE 1:**
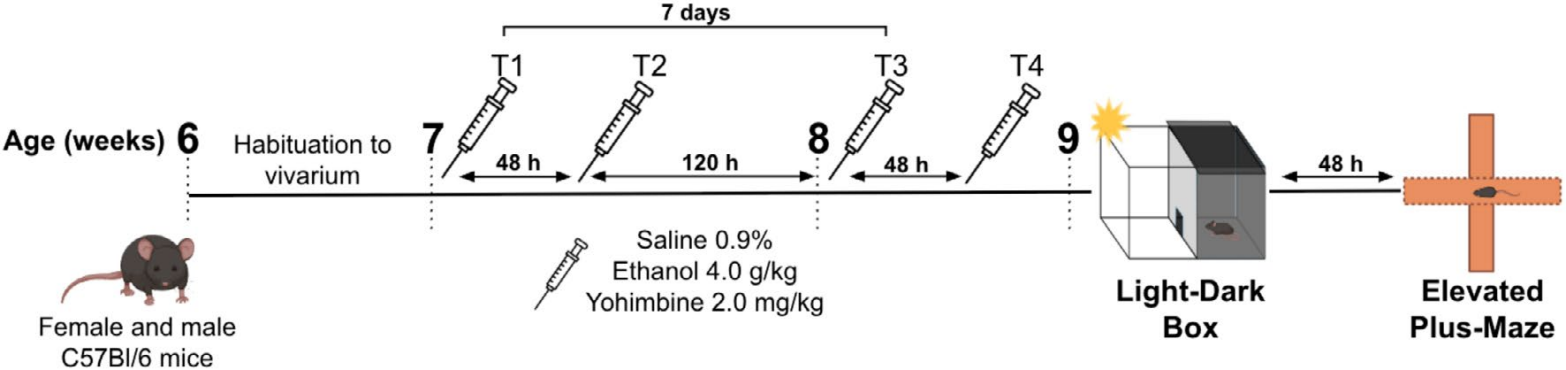
Experimental timeline of the study. Male and female C57BL/6J mice were habituated to the vivarium at 6 weeks of age and then received four intraperitoneal injections (T1‐T4) over a 2‐week period starting at 7 weeks of age. Animals were treated with saline (0.9%), ethanol (4.0 g/kg), or yohimbine (2.0 mg/kg). The first two injections were separated by 48 h, the third injection occurred after a 120 h interval, and the fourth injection was administered 48 h later. At 9 weeks of age, mice were tested in the Light–Dark Box (LDB) to assess anxiety‐like and exploratory behavior, followed 48 h later by the Elevated Plus Maze (EPM). Numbers above the timeline indicate the age of the animals (in weeks) at each stage of the protocol.

Mice underwent four administrations of saline (Ctrl; 0.9% w/v; Samtec, Ribeirao Preto, SP, Brazil), ethanol (EtOH; 4.0 g/kg, i.p., 20% v/v in 0.9% saline; from 95% ethanol, 01A1082.01.BJ, Labsynth, Diadema, SP, Brazil), or yohimbine (Yoh; 2.0 mg/kg, i.p., 0.08 mg/mL in 0.9% saline; from ≥ 98% yohimbine hydrochloride, Y3125, Sigma‐Aldrich, Munich, BY, Germany), an α2‐adrenergic receptor antagonist which increases noradrenaline release and activates the HPA axis (Langer 1980; Tanaka et al. 2000), over the course of 2 weeks (during the 7^th^ and 8^th^ weeks of age), with a minimum interval of 48 h between injections (see Figure 1 for detailed protocol). Injections were administered between 9:00 a.m. and 12:00 p.m. We selected this exposure protocol based on our previous work (Pichinin et al. 2026) and on practical considerations related to administering high ethanol doses. In protocols using high doses of ethanol (e.g., 3–4 g/kg), daily administration can substantially increase the risk of adverse effects such as severe sedation and hypothermia, which may lead to increased mortality. Therefore, we implemented a schedule with four intoxication episodes over 2 weeks, with a minimum interval of 48 h between administrations, to ensure animal safety. At the same time, this spacing allowed us to model repeated heavy intoxication episodes, which more closely resemble the intermittent pattern of high‐intensity drinking commonly observed during adolescence.

### 2.3 Blood Ethanol Concentration

Blood samples were collected from a separate cohort of C57BL/6 mice, distinct from those later used in the LDB and EPM experiments. Samples were obtained 30 min after i.p. injection on the first (T1) and last (T4) days of drug protocol via submandibular vein puncture using a lancet (Golde et al. 2005). Blood was stored in tubes containing ethylenediaminetetraacetic acid (EDTA; 60 mg/mL), centrifuged at 2300 rpm at 4°C for 15 min, and the supernatant (plasma) was collected and stored at −20°C. Blood ethanol concentration (BEC) analysis was performed at the Laboratory of Toxicological Information and Assistance Center (CIATox) of the Universidade de Campinas (Unicamp), using gas chromatography with a flame ionization detector (GC‐FID 2010 Plus, Shimadzu, Japan) coupled to a headspace autosampler (HS‐10, Shimadzu, Tokyo, Japan). The chromatographic separation was performed on a DB‐BAC1 UI chromatographic column (30 m × 0.320 mm × 1.8 μm; Agilent Technologies, USA), maintained at 40°C (isothermal), with a column equilibration time of 1 min. The injection block parameters were: injector temperature at 150°C, injection mode in split mode 1:40, carrier gas pressure at 60.5 kPa, total flow of 85 mL/min, column flow of 2 mL/min, linear velocity at 33.7 cm/s, purge flow of 3 mL/min, and flow control mode in linear velocity. The flame ionization detector parameters were: detector temperature at 250°C, flame composed of hydrogen (40 mL/min), synthetic air (400 mL/min), and makeup gas (nitrogen, 30 mL/min). The total run time was 5 min. The autosampler parameters were: oven temperature of 80°C, transfer line temperature of 105°C, sample line temperature of 90°C, pressurized gas pressure of 100 kPa, shaking level of 2, shaking time of 1 min, shaking equilibre time of 0.5 min, equilibrating time of 4 min, pressurizing time of 1 min, pressurization equilibre time of 0.17 min, load time of 0.5 min, load equilibre time of 0.17 min, injection time of 1 min, and GC cycle time of 5.6 min. The test detection range was 0.05 to 5.0 g/L.

### 2.4 Behavioral Procedures

We employed two well‐established paradigms to assess anxiety‐like behavior in rodents: the LDB and the EPM. The LDB consisted of an apparatus with two compartments: one highly illuminated (~400 lx) (Takao and Miyakawa 2006), and the other mostly dark (~3 lx) side. Compartments measured the same size (22 × 24 × 26 cm), and were connected by a guillotine door. The animal was placed in the dark compartment with the guillotine door closed, which was opened after 5 s to start the test. It is important to point out that the LDB protocol used in this work was slightly different from classic protocols, which involve a smaller dark side and initiating the test with the animal in the light side (Bourin and Hascoët 2003; Crawley and Goodwin 1980).

Our EPM consisted of a wooden apparatus, fully covered in waterproof varnish to prevent urine impregnation, elevated 50 cm above the ground, with two open and two closed (protected) arms. The open and closed arms equally measured 27.8 cm and the height of closed arm walls was 14 cm. Arms were connected by a square central area (7.8 × 7.8 cm). Luminosity reached ~46 and ~16 lx in the open and closed arms, respectively, similar to previous reports (Bruchas et al. 2009; Pichinin et al. 2026; Tseitlin et al. 2023; Yu et al. 2007). The test started with the animal placed in the center of the maze, facing one of the open arms.

Behavioral testing initiated 5–7 days after the last drug exposure. Animals were acclimated to the experiment room for 30 min before testing. The LDB and EPM tests were performed during the light phase of the light/dark cycle (between 12:00 and 3:00 p.m.). Tests were recorded using a webcam (Logitech C920, 30 fps) and streamed to a computer in an adjacent room. Each mouse was tested only once on each apparatus, and the apparatuses were cleaned with a 20% ethanol solution between sessions. After each session, animals were immediately placed back in their homecage. Animals were tested in the LDB and, 48 h later, in the EPM. In both tests, animals were allowed to explore the apparatus for 5 min.

During this time, the duration and frequency of behaviors, including time spent in each compartment, number of risk assessment behaviors, entries in each compartment, as well as other behaviors listed in Table S1, were recorded. Risk assessment behaviors were evaluated into two categories: NoGo behaviors, in which the animal, right after evaluating the risk, does not enter the aversive compartment, and Go behaviors, in which the animal decides to explore the aversive environment after risk assessment (Pichinin et al. 2026). It is important to note that, due to limitations in our setup, LDB behaviors were recorded only on the light side. Additionally, during the EPM testing, a power outage interrupted the recording of one male animal from the ethanol group, resulting in the loss of the behavioral data. Consequently, this animal was excluded from the EPM analysis.

### 2.5 Data Exportation and Statistical Analyses

Recorded videos of the LDB and EPM were analyzed using BORIS (v.8.21.8), an open‐source software for behavior analysis (Friard and Gamba 2016). All behaviors listed in Table S1 were quantified by the experimenter, who was blinded to the experimental groups. Data were exported and processed using a modified version of a custom‐made Python (v.3.7.6) code (available at GitHub). Locomotion (distance and velocity) during the EPM test was assessed using ezTrack, an open‐source video analysis pipeline (Pennington et al. 2019). Go and NoGo risk assessment indexes were calculated by summing the number of Go and NoGo head‐outs and stretches, respectively, for each animal.

The number of discrete behaviors or time spent in any compartment were compared among groups using Generalized Linear Models (GzLM), with Poisson and linear distributions applied as appropriate. Linear Mixed Models (LMM) were used to compare BEC levels between the first and last treatment days, with sex and treatment day as factors.

Behavioral sequences were analyzed using a first‐order Markov chain framework to quantify the global contribution of specific behavioral transitions to the animals' overall behavioral repertoire, as in Pichinin et al. (2026). For both LDB and EPM transition analyses, data from female and male mice were pooled, as sex did not significantly affect the majority of behavioral variables analyzed. Because standardized statistical approaches for directly comparing full behavioral transition matrices across multiple groups are limited, particularly when some transitions are sparse, we focused our statistical analyses on first‐order transition probabilities. For each animal, the probability of occupying each behavioral state (p(A)) was computed based on the relative frequency of that behavior across the session. To capture both state occupancy and sequential organization across the entire behavioral chain, for each pair of behaviors A→B, 1^st^ order transition probabilities were computed by multiplying the probability of occupying the initial state by the probability of observing the subsequent behavior, yielding a joint probability metric of p(A) × p(A→B). Group‐level comparisons were therefore conducted using pairwise χ^2^ tests on 1^st^ order transition probability, comparing the control group with each experimental group (Ctrl vs. EtOH; Ctrl vs. Yoh), as defined a priori to address the study's primary hypotheses regarding the effects of alcohol exposure and pharmacological stress on behavioral organization. In line with previous studies (Espejo 1997; Lino de Oliveira et al. 2005), state events occurring less than 1% of the time and transitions with less than 2% occurrence were excluded from the analyses.

To assess relationships between variables, we ran correlation analyses. A Shapiro–Wilk normality test was first performed and, because not all variables followed a normal distribution, Spearman's rank correlation was used. Analyses were conducted with animals separated into experimental groups to determine whether relationships between variables were group‐dependent. Correlations were performed without considering sex, as this factor did not have a significant effect on the majority of variables analyzed using GzLM.

Markov chains were built using Keynote (v. 14.5; 7045.0.17), with circles proportional to the frequency of each behavior, and arrow transparency proportional to transition probabilities. The numbers in the graphs represent 1^st^ order transition probabilities (p(A) x p(A→B)). Bonferroni post hoc tests were applied when necessary, and a *p*‐value < 0.05 was considered statistically significant. Statistical analyses were performed using Jamovi (v.2.4.8.0) and graphs were assembled in Graphpad Prism (v.10.1.1). Data are presented as the mean ± standard error of the mean (SEM).

## 3 Results

### 3.1 Ethanol Administration Induces High Levels of Intoxication

**FIGURE 2:**
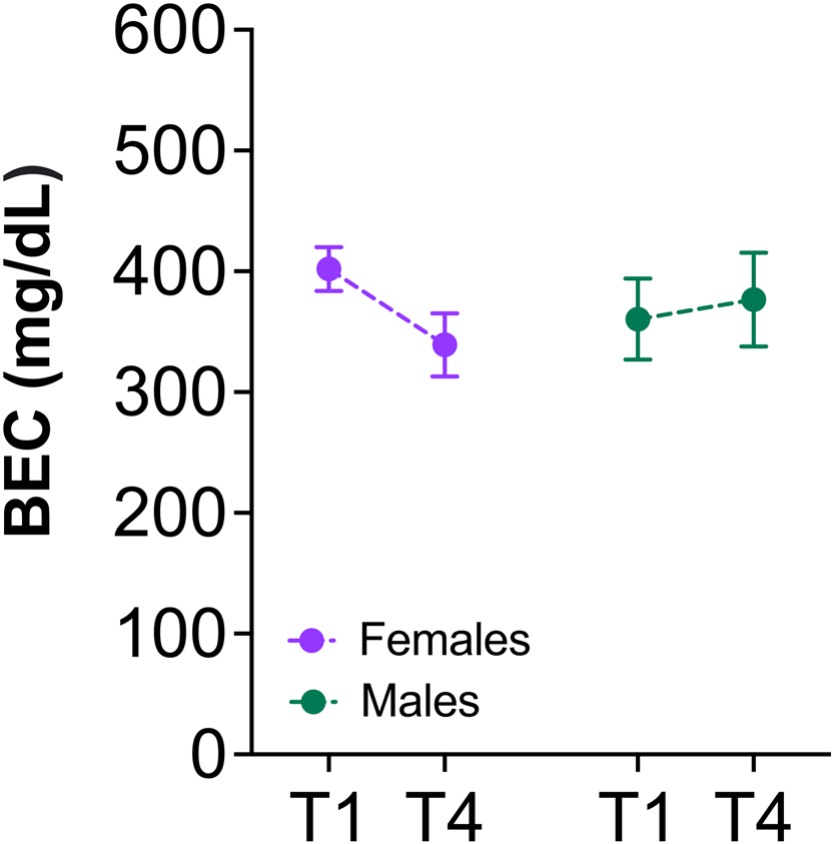
Blood EtOH Concentrations (BEC) 30 min after i.p. administration on the first (T1) and last (T4) days of the protocol. Mice presented similar BEC levels on the first and last treatment days. Sex was not significant. Sex‐separated analyses showed similar BEC levels on the first and last treatment days in females and males. *n* = 6/sex. Data are presented as mean ± SEM.

Ethanol (4.0 g/kg) induced high levels of BEC that remained stable across treatment days (F_(1,4)_ = 0.69) and did not differ between females and males (F_(1,4)_ = 0.03; Figure 2). Sex‐separated analyses showed similar levels of BEC on the first and last treatment days in females (F_(1,2)_ = 1.60) and males (F_(1,2)_ = 3.00).

### 3.2 Ethanol and Yohimbine Exposures During Late Adolescence Impair Risk Assessment Behaviors in Adulthood in Both Female and Male Mice

**FIGURE 3:**
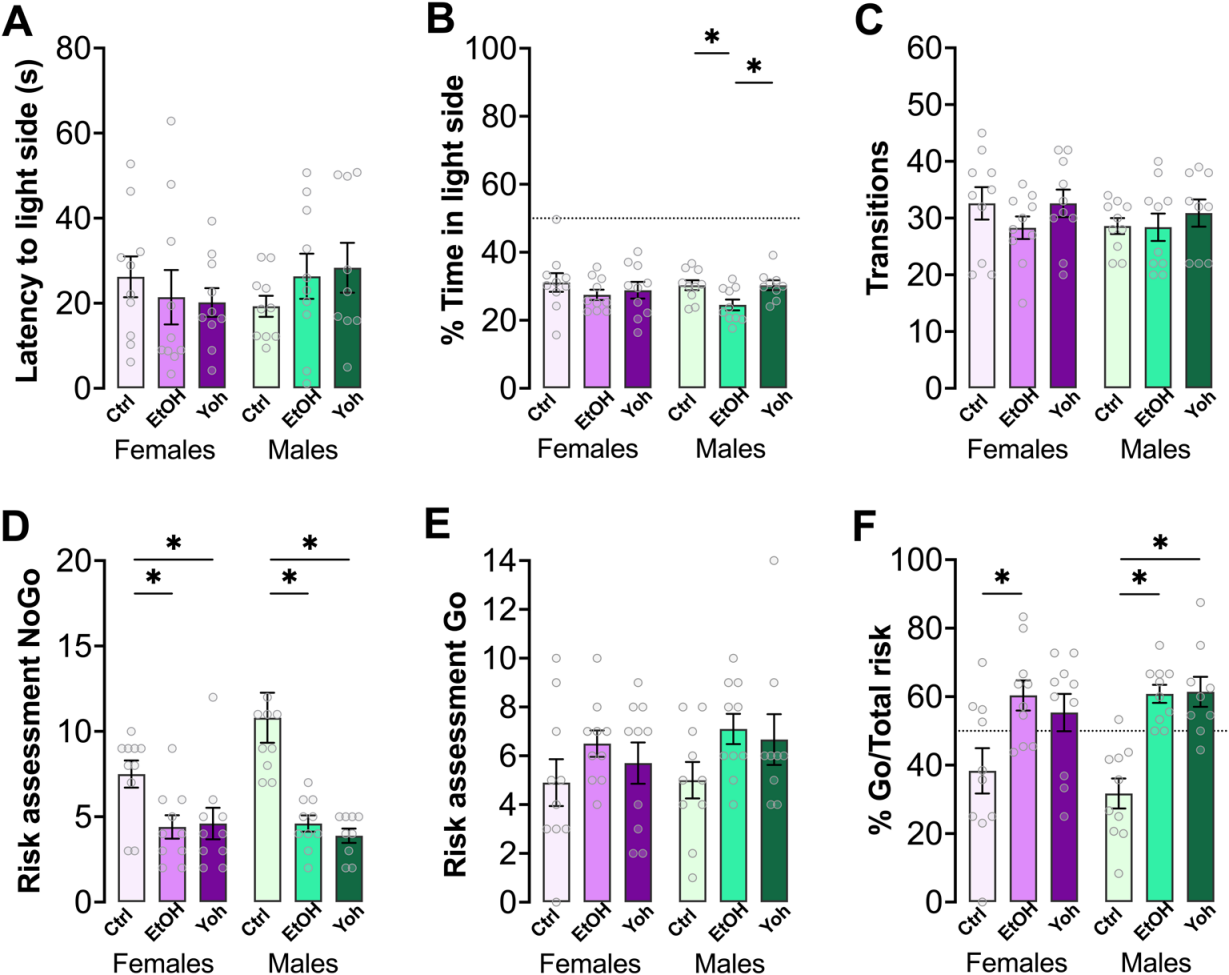
Anxiety‐like, exploratory and risk assessment behaviors of adult female and male mice (9 weeks old) in the LDB 5 days after saline (Ctrl), ethanol (EtOH) or yohimbine (Yoh) exposures during late adolescence. (A) Latency to pass to the light side was similar in both sexes, and sex‐separated analyses showed no group effect. (B) Percentage of time in the light side was similar in both sexes. Sex‐separated analyses showed that males in the EtOH group spent less time in the light side compared to Ctrl and Yoh. No significant group differences were found in females. (C) Transitions were similar in both sexes, and sex‐separated analysis showed no effect of group. (D) NoGo risk assessment analysis showed no significant effect of sex, and sex‐separated analysis showed reduced NoGo risk assessment behaviors in the EtOH and Yoh groups in comparison to controls in female and male mice. (E) Go risk assessments were similar in both sexes, and sex‐separated analyses showed no significant group effect. (F) The percentage of Go risk assessment among total risk assessment behaviors was similar in both sexes. Sex‐separated analysis showed that EtOH females more frequently transitioned to the light side, and a similar effect was observed in EtOH and Yoh males. The dashed line represents 50%, indicating an equal distribution between Go and NoGo risk assessment. *p < 0.05; Ctrl: *n* = 10/sex; EtOH: *n* = 10/sex; Yoh: *n* = 10 females, 9 males. Data are presented as mean ± SEM.

To test whether ethanol intoxication or stress exposure during late adolescence (7–8 weeks of age) could affect behavior in early adulthood (9 weeks of age), mice were tested in the LDB 5 days after the last drug exposure. Latency to pass to the light side was similar between female and male mice (χ^2^_(1)_ = 0.37), and sex‐separated analyses showed no group effect (females: χ^2^_(2)_ = 0.80; males: χ^2^_(2)_ = 2.06) (Figure 3A). The percentage of time in the light side was similar in both sexes (χ^2^_(1)_ = 0.24). Sex‐separated analyses revealed no group differences in the percentage of time spent in the light side among female mice (χ^2^_(2)_ = 1.28), but ethanol‐treated male mice spent less time in the light in comparison to controls and yohimbine‐treated mice (χ^2^_(2)_ = 10.10, *p* = 0.006; EtOH vs. Ctrl: *p* = 0.03; EtOH vs. Yoh: *p* = 0.035) (Figure 3B). These results indicate that late adolescence ethanol intoxication episodes, but not yohimbine exposure, induce a significant anxiogenic behavior in males but not in females. No effects on transitions between the dark and light sides were observed between female and male mice (χ^2^_(1)_ = 1.74), and no significant group difference was observed in sex‐separated analyses (females: χ^2^_(2)_ = 4.02; males: χ^2^_(2)_ = 1.21) (Figure 3C), although intoxication with ethanol influenced other exploratory behaviors in the LDB. For instance, ethanol‐treated males performed less rears compared to the other groups (χ^2^_(2)_ = 11.5, *p* = 0.003; EtOH vs. Ctrl: *p* = 0.009, EtOH vs. Yoh: *p* = 0.011). Sniffs and other exploratory behaviors were not affected by ethanol or yohimbine exposure (Figure S1A–C).

A central goal of this study was to determine whether prior alcohol intoxication or stress exposure can promote risky decision‐making later in life. To address this, we quantified risk assessment behaviors, distinguishing between NoGo responses and Go responses. NoGo risk assessment analysis showed no significant effect of sex (χ^2^_(1)_ = 0.58). Ethanol and stress induced fewer NoGo risk assessments when compared to control mice in both sexes (females: χ^2^_(2)_ = 10.40, *p* = 0.005; EtOH vs. Ctrl: *p* = 0.015, Yoh vs. Ctrl: *p* = 0.027; males: χ^2^_(2)_ = 40.90, *p* < 0.001; EtOH vs. Ctrl: *p* < 0.001, Yoh vs. Ctrl: p < 0.001) (Figure 3D). On the other hand, Go risk assessment was not affected by prior treatment in both sexes (females: χ^2^_(2)_ = 2.25; males: χ^2^_(2)_ = 4.04) (Figure 3E). To better characterize how often animals chose the risky option (Go decisions), we compared the proportion of Go risk assessment events relative to the total number of risk‐assessment behaviors (Figure 3F). There was no significant effect of sex (χ^2^_(1)_ = 0.00). Control female mice predominantly returned to the dark side after assessing risk, whereas ethanol‐treated mice more frequently transitioned to the light side (χ^2^_(2)_ = 8.63, *p* = 0.013; EtOH vs. Ctrl: *p* = 0.028). A similar pattern was observed in males, in which both ethanol‐ and yohimbine‐treated animals showed a higher proportion of Go events compared with controls (χ^2^_(2)_ = 39.40, *p* < 0.001; EtOH and Yoh vs. Ctrl: *p* < 0.001).

**FIGURE 4:**
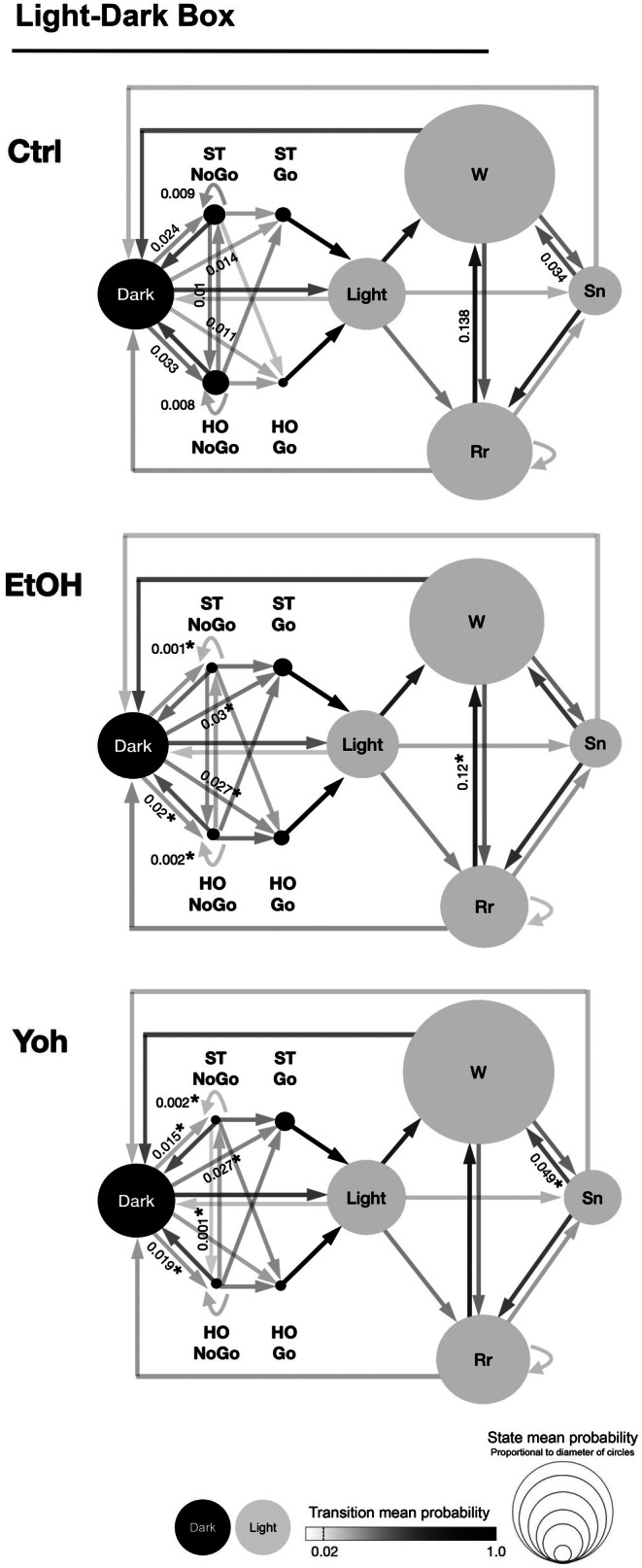
Markov chains representing the sequencing and chaining of behavioral events in the experimental groups during the LDB test. Black circles represent behaviors in the dark side and gray circles represent behaviors in the light side. Circles' sizes are proportional to the number of occurrences of each behavior, while the arrows' color gradient represents the probability of each transition. Numbers represent 1^st^ order transition probabilities. For experimental groups, only significant difference from Ctrl are displayed, with asterisks indicating *p* < 0.05. Complete statistics for these transitions are listed in Table S2. HO: Head‐out, St: Stretch, Rr: Rear, Sn: Sniff, W: Walk. Ctrl: *n* = 20 (10/sex); EtOH: *n* = 20 (10/sex); Yoh: *n* = 19 (10 females, 9 males).

To analyze 1^st^ order transition probabilities within the ethogram, we constructed Markov chains for each group illustrating the resulting behavioral structure during the LDB test (Figure 4). For this analysis, data from male and female mice were pooled, as frequentist analyses of discrete behaviors revealed no robust sex‐dependent differences, and pooling increased both statistical power and the stability of transition probability estimates. Briefly, animals exposed to ethanol and yohimbine showed significantly lower transitions probability from the dark compartment to NoGo risk assessment behaviors compared to controls (i.e., dark entry—NoGo head‐out, EtOH vs. Ctrl: χ^2^_(1)_ = 9.63; *p* = 0.002; Yoh vs. Ctrl: χ^2^_(1)_ = 7.94; *p* = 0.005). In contrast, these animals exhibited increased transition probability toward Go risk assessment behaviors (i.e., dark entry—Go stretch, χ^2^_(1)_ = 8.12; EtOH vs. Ctrl: *p* = 0.004; Yoh vs. Ctrl: χ^2^_(1)_ = 6.56; *p* = 0.010 and dark entry—Go head‐out, EtOH vs. Ctrl: χ^2^_(1)_ = 9.76; *p* = 0.002), indicating a shift toward less cautious exploratory behavior. Please refer to Table S2 for a complete list and statistical description of significant differences in 1^st^ order transition probabilities between the EtOH and Yoh groups compared with Ctrl.

**FIGURE 5:**
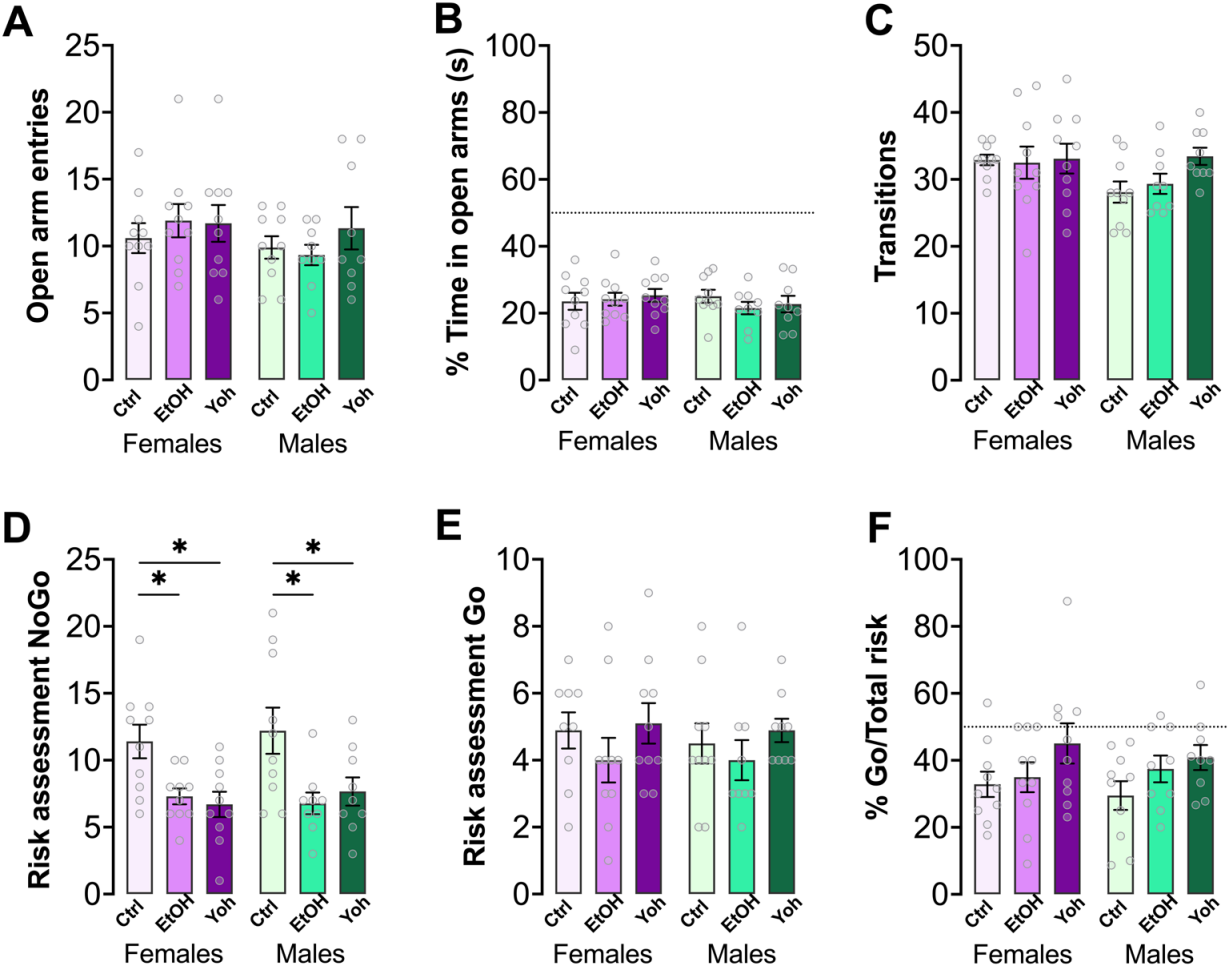
Anxiety‐like, exploratory and risk assessment behaviors of adult female and male mice (9 weeks old) in the EPM 7 days after saline (Ctrl), ethanol (EtOH) or yohimbine (Yoh) exposures during late adolescence. (A) Number of open arm entries was similar in both sexes, and sex‐separated analyses showed no group effects. (B) Percentage of time in the open arms was similar in both sexes, and sex‐separated analyses showed no group effects. (C) Transitions were similar in both sexes, and sex‐separated analyses showed no group effects. (D) NoGo risk assessment was similar in both sexes, and sex‐separated analysis showed similar group effects in females and males. (E) Go risk assessment was similar in both sexes, and sex‐separated analyses showed no group effects. (F) The percentage of Go risk assessment among total risk assessment behaviors was similar in both sexes, and sex‐separated analysis did not point to significant group effects. The dashed line represents 50%, indicating an equal distribution between Go and NoGo risk assessment. Ctrl: *n* = 10/sex; EtOH: *n* = 10 females, 9 males; Yoh: *n* = 10 females, 9 males. Data are presented as mean ± SEM.

Seven days after the last drug exposure, and 2 days after LDB, animals were tested on the EPM. Anxiety‐like and exploratory behaviors in the EPM were not affected by ethanol and yohimbine exposures during late adolescence. There was no significant effect of sex on open arm entries (χ^2^_(1)_= 1.75) or percentage of time spent in the open arms (χ^2^_(1)_= 0.42). Sex‐separated analyses also revealed no significant group effects for these variables (open arm entries: females: χ^2^_(2)_ = 0.87; males: χ^2^_(2)_ = 1.86 ‐ Figure 5A; percentage of time in the open arms: females: χ^2^_(2)_ = 0.34; males: χ^2^_(2)_ = 1.50 ‐ Figure 5B). Transitions between light and dark sides were also not affected by sex (χ^2^_(1)_ = 2.71), and did not differ between groups in either sex (females: χ^2^_(2)_ = 0.06; males: χ^2^_(2)_ = 4.75; Figure 5C). Additionally, there was no significant effect of group on distance (χ^2^_(2)_ = 0.64) and velocity (χ^2^_(2)_ = 0.53) throughout EPM testing (Figure S3A,B). Additional exploratory measures were largely similar across groups (Figure S2), but females displayed higher frequencies of rearing than males (χ^2^_(1)_ = 4.35, *p* = 0.037), and head dips did not differ between sexes (χ^2^_(1)_ = 2.75) or treatments (females: χ^2^_(2)_ = 0.29; males: χ^2^_(2)_ = 2.75; Figure S2E,F) (Figure S2A–F).

Regarding risk assessment variables, there was no significant sex effect on NoGo risk assessment (χ^2^_(1)_ = 0.13), and sex‐separated analyses showed that both ethanol and yohimbine reduced the frequency of NoGo risk assessment behaviors similarly in females (χ^2^_(2)_ = 14.80, *p* < 0.001; EtOH vs. Ctrl: *p* = 0.009, Yoh vs. Ctrl: *p* = 0.002) and males (χ^2^_(2)_ = 17.50, *p* < 0.001; EtOH vs. Ctrl: *p* < 0.001; Yoh vs. Ctrl: *p* = 0.006; Figure 5D). Go risk assessment behaviors were not affected by sex (χ^2^_(1)_ = 0.38) and were similar in all groups, according to sex‐separated analyses (females: χ^2^_(2)_ = 2.80; males: χ^2^_(2)_ = 1.76; Figure 5E). Additionally, there was no effect of sex (χ^2^_(1)_ = 0.12) or group on the proportion of Go risk assessment behaviors among total risk assessment behaviors (females: χ^2^_(2)_ = 3.62; males: χ^2^_(2)_ = 4.26; Figure 5F).

**FIGURE 6:**
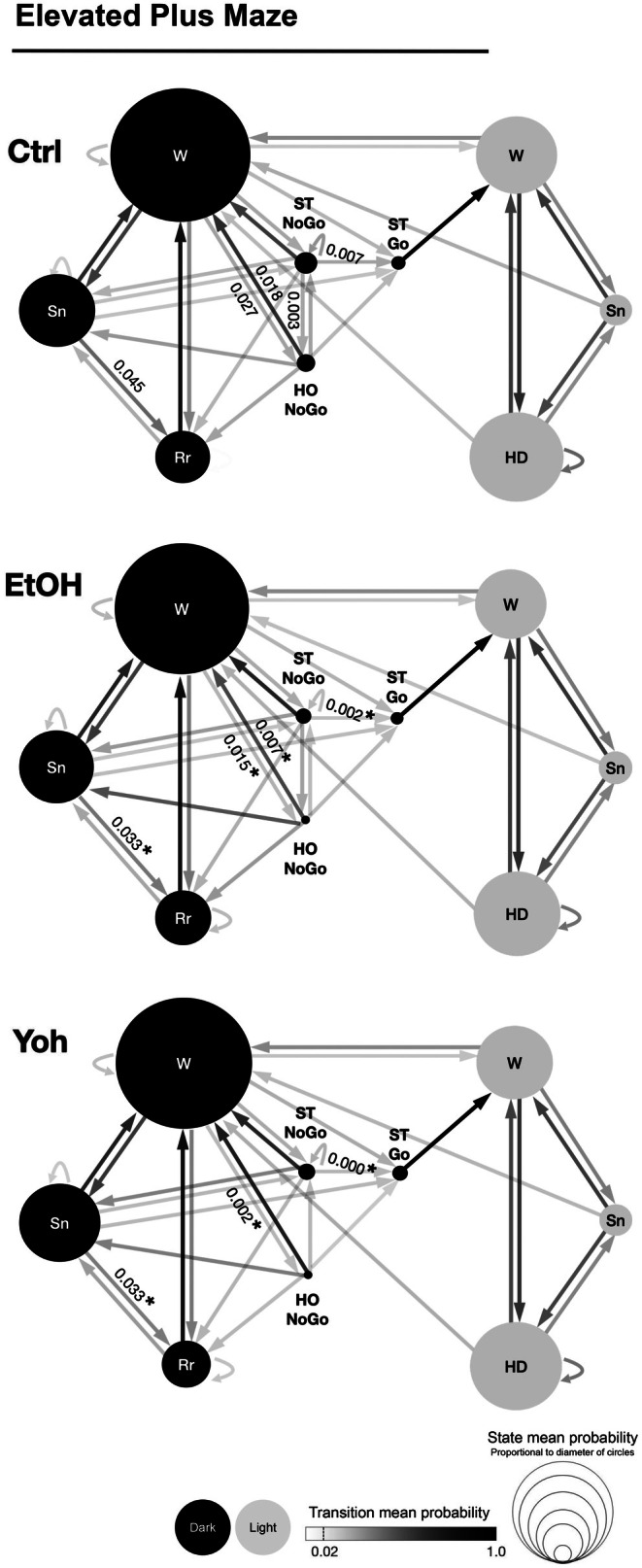
Markov chains representing the sequencing and chaining of behavioral events in the experimental groups during the EPM test. Black circles represent behaviors in the closed arms and gray circles represent behaviors in the open arms of the maze. Circles' sizes are proportional to the number of occurrences of each behavior, while the arrows' color gradient represents the probability of each transition. Numbers represent transition 1^st^ order transition probabilities. For experimental groups, only significantly difference from Ctrl are displayed, with asterisks indicating *p* < 0.05. Complete statistics for these transitions are listed in Table S3. HO: Head‐out, St: Stretch, Rr: Rear, Sn: Sniff, W: Walk. Ctrl: *n* = 20 (10/sex); EtOH: *n* = 19 (10 females, 9 males); Yoh: *n* = 19 (10 females, 9 males).

To examine the structural organization of behaviors in the EPM, we analyzed transition probabilities between exploratory and risk assessment behaviors and constructed Markov chains summarizing the behavioral structure of the task (Figure 6). As for the LDB, data from male and female mice were pooled based on the lack of robust significant differences shown by frequentist analyses of discrete behaviors. Ethanol‐ and yohimbine‐exposed animals exhibited fewer transitions from walking in the closed arms to NoGo head‐outs compared to controls (EtOH vs. Ctrl: χ^2^₍₁₎ = 5.80; *p* = 0.016; Yoh vs. Ctrl: χ^2^₍₁₎ = 10.36; *p* = 0.001), indicating a reduced engagement in cautious risk assessment from protected areas. Additionally, experimental groups showed lower transition probabilities from sniffing to rearing (both in closed arms) relative to controls (EtOH vs. Ctrl: χ^2^₍₁₎ = 5.73; *p* = 0.017; Yoh vs. Ctrl: χ^2^₍₁₎ = 4.23; *p* = 0.04), which is consistent with diminished vertical exploration. Other significant differences from EtOH and Yoh groups relative to Ctrl are listed in Table S3. Together, these alterations suggest that ethanol and yohimbine modify the structure of behavior in the EPM, altering how exploratory and risk assessment events are sequentially organized.

**FIGURE 7:**
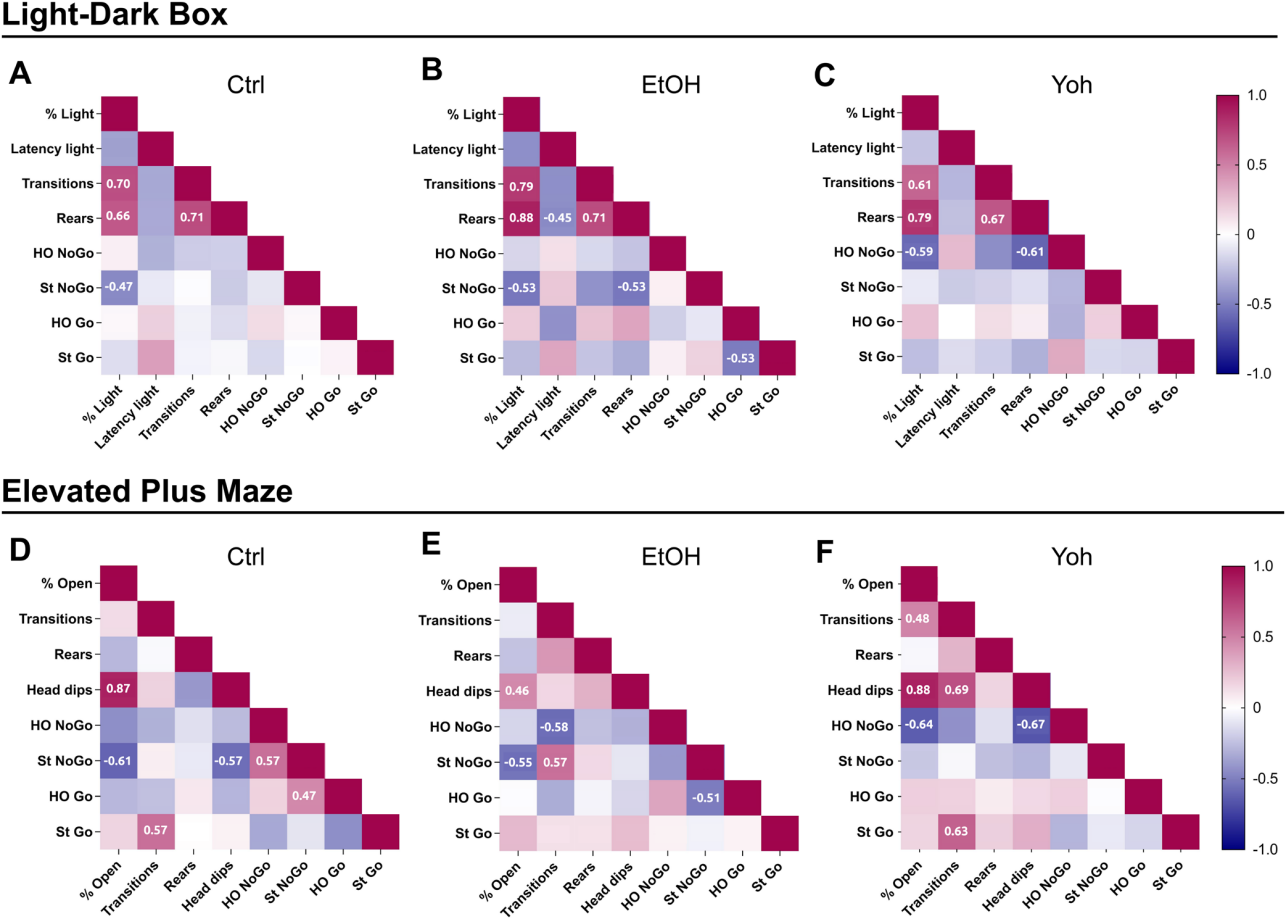
Spearman's correlation heat maps for female and male pooled data from the LDB and EPM tests, respectively. Heatmaps depict Spearman's rank correlation coefficients (ρ) between exploratory and risk assessment behaviors for control (Ctrl), ethanol‐ (EtOH), and yohimbine‐exposed (Yoh) mice. Numerical values shown within the cells correspond to statistically significant correlations only (Spearman's ρ, *p* < 0.05). Color intensity reflects the strength and direction of the correlation. Autocorrelated variables and non‐significant correlations are left blank. Data from male and female mice were pooled for correlation analyses.

To further characterize how individual behavioral measures relate to one another beyond transition structure, we performed pairwise correlations across behavioral variables from all mice. In the LDB, the control group showed correlations between classical anxiety‐related measures (e.g., percentage of time in the light compartment) and exploratory behaviors, whereas risk assessment measures were more weakly associated with exploratory variables. Drug exposure altered these relationships, with ethanol and yohimbine producing distinct correlation patterns among anxiety‐like, exploratory, and risk assessment behaviors (Figure 7A–C). EPM correlation analyses revealed that control animals exhibited strong positive correlations between exploratory behaviors, such as head dips, and anxiety‐like behavior, represented by the percentage of time in the open arms. Notably, NoGo risk assessment was negatively correlated to head dips (Figure 7D). In ethanol‐exposed mice, this pattern was altered, with a weakened association between exploratory and anxiety‐like behaviors (Figure 7E). In contrast, yohimbine‐treated animals displayed stronger positive correlations between exploratory and anxiety‐like measures, including transitions, head dips, and percentage of time in the open arms, as well as stronger negative correlations between NoGo risk assessment (particularly NoGo head‐outs), and both anxiety‐like and exploratory behaviors (Figure 7F). Together, these findings suggest that ethanol and yohimbine differentially affect the coordination between different types of behavior. While ethanol exposure weakens the coordination between exploratory and anxiety‐like behaviors, suggesting a dissociation of these processes, yohimbine strengthens their association, particularly with risk assessment measures, indicating that these behaviors are more strongly associated following yohimbine repeated exposure.

## 4 Discussion

The present study demonstrates that repeated ethanol intoxication or pharmacological stress during late adolescence produces alterations in risk assessment behavior in early adulthood. Importantly, such effects occurred in the absence of major changes in classical anxiety‐like measures, suggesting a dissociation between anxiety and the sequential organization of approach‐avoidance behaviors. These results indicate a bias toward less cautious decision‐making when animals previously exposed to ethanol and yohimbine are presented with new, potentially threatening environments.

Risk assessment, which is a defensive strategy animals use to balance exploration of unfamiliar environments with the need to avoid potential threats, is reliably evoked in approach‐avoidance paradigms such as the LDB and the EPM (Augustsson and Meyerson 2004), and can be modulated independently of general anxiety levels, providing information that is not captured by traditional measures such as time spent in open arms or illuminated compartments. In the present study, exposure to high doses of ethanol or the *α*2‐adrenergic antagonist yohimbine during late adolescence biased animals toward risk‐taking behaviors in adulthood, as reflected in decreases of NoGo risk assessment, with no major effects on general exploration or anxiety‐like behaviors. These results suggest a diminished tendency to withhold action following risk assessment, reflecting a shift toward riskier decision strategies.

Alcohol exposure has been widely associated with impaired inhibitory control and increased impulsivity in humans and rodents (Reynolds et al. 2006; Weafer et al. 2021), indicating that alcohol impairs the ability to suppress actions (which involves inhibitory control). Additionally, a meta‐analysis by McPhee and Hendershot (2023) further confirmed that acute alcohol consistently impairs the ability to withhold or stop actions, with stronger effects at higher intoxication levels. In rodents, ethanol administrations during adulthood induced impairments in reversal learning that were evident during abstinence (5–7 days after last exposure), indicative of persistent behavior despite a change in contingency (Badanich et al. 2016). These findings support the notion that repeated alcohol impairs inhibitory control and, consequently, risk assessment, providing a framework to interpret the reductions in NoGo behaviors observed in the present study.

Stress exposure has also been associated with alterations in risk evaluation. Both human and animal studies indicate that stress can increase impulsive decision‐making and promote risk‐taking behaviors (Roos et al. 2017; Shafiei et al. 2012). Pharmacological stressors such as yohimbine increase risky choice in risk discounting tasks when administered shortly before testing, an effect linked to heightened catecholaminergic signaling within prefrontal circuits (Münster et al. 2024). By blocking α2‐adrenergic autoreceptors, yohimbine enhances noradrenergic tone in limbic and prefrontal regions involved in arousal, emotional processing, and behavioral inhibition, promoting anxiogenic‐like states and reduced cautious evaluation (Blanchard et al. 1993; Sperl et al. 2022; Tanaka et al. 2000; Vasa et al. 2009). These observations are consistent with the reduction in NoGo risk assessment behaviors observed in the present study. Additionally, our results align with previous findings showing an increased proportion of open arm entries following stretch‐attend postures in the closed arms of the EPM in rats exposed to chronic social stress (Stickling and Rosenkranz 2025).

Age, sex, and dosage are critical biological and procedural variables shaping the long‐lasting effects of alcohol or stress exposure. Adolescence represents a particularly critical developmental window for the maturation of neural circuits supporting behavioral inhibition and decision‐making. Consistent with this, alcohol exposure during this period produces lasting alterations in risk‐related strategies and inhibitory control that extend into adulthood (Towner and Varlinskaya 2020). Previous work from our group has indicated that a repeated moderate dose of ethanol (3.2 g/kg) during early adolescence (weeks 5–6) selectively decreases NoGo risk assessment in adult male mice tested in the LDB (Pichinin et al. 2026). Extending these findings to late adolescence (weeks 7–8), the present study shows that repeated higher‐dose ethanol exposure, as well as pharmacologically induced stress, reduces NoGo risk assessment in adult male and female mice in both the LDB and EPM. Thus, late adolescence may represent a particularly vulnerable period for disruptions in inhibitory control, resulting in a stronger tendency toward exploration and risk‐taking. These results align with previous findings showing that intermittent ethanol exposure during late adolescence, at lower (2.0 g/kg) and more frequent doses than the one used in the present study, leads to lasting and timing‐dependent disruptions in attention, impulsivity, and risky decision‐making in mice (Sanchez‐Roige et al. 2014). Our results add to this by showing that not only ethanol, but also yohimbine, reduce cautious risk evaluation, pointing to overlapping vulnerabilities in behavioral inhibition and decision‐making circuits.

Beyond changes in the frequency of behaviors, our findings indicate that adolescent ethanol and stress exposures also alter how exploratory and risk assessment behaviors are sequentially organized. Using transition‐based analyses and Markov chain modeling, we observed that ethanol‐ and yohimbine‐treated animals exhibited reorganized behavioral sequences during exploration in both the LDB and the EPM. In the LDB, these alterations were characterized by reduced transitions from the protected dark compartment to NoGo risk assessment behaviors and a relative bias toward transitions leading to Go behaviors, indicating a shift in how animals engage with and act upon potentially dangerous, aversive zones. In the EPM, a complementary pattern emerged, with ethanol‐ and yohimbine‐treated animals showing fewer transitions from walking in the closed arms to NoGo head‐outs, alongside altered exploratory sequences involving sniffing and rearing. Notably, these effects reflect changes in behavioral structure rather than just increases or decreases in individual behaviors, highlighting that decision‐making alterations may be embedded in the temporal, sequential flow of actions.

Some studies have reported correlations between risk assessment and anxiety‐like behaviors in rodents. For example, increased anxiety‐like behavior in the EPM has been associated with elevated risk assessment (Ohl et al. 2001), and anxiolytic drugs reduce risk assessment behaviors on the same test and also in the LDB (Cruz et al. 1994; Griebel et al. 1998). However, risk assessment behaviors can be present regardless of the level of anxiety, reflecting partially dissociable defensive strategies. In line with this view, our results indicate that adolescent ethanol and stress exposure altered the structural integration of behavioral components rather than simply their frequency. Across both behavioral tests, ethanol and yohimbine produced a marked reorganization of the correlational structure of behavior linking exploratory, anxiety‐like, and risk assessment behaviors relative to controls, revealing test‐specific alterations in behavioral integration. While in both tests control animals exhibited a balanced pattern in which risk assessment constrained exploration in anxiogenic environments (Ohl et al. 2001; Rodgers and Dalvi 1997), ethanol‐ and yohimbine‐exposed mice showed disrupted relationships between these behavioral domains. For example, ethanol strengthened associations among exploratory behaviors while decoupling NoGo risk assessment from exploratory and anxiety‐like measures, whereas yohimbine treatment selectively reinforced negative associations between exploration and NoGo risk assessment. Together, these findings support the idea that classical measures of anxiety do not fully capture the organization of defensive behavior, and that sequential and structural analyses can reveal alterations that remain undetected using traditional summary measures.

Some limitations to our study should be acknowledged. Although both male and female mice were included and analyzed for the frequentist data set, the Markov chain analyses of behavioral sequences were conducted using pooled data due to lack of strong differences on alcohol and yohimbine effects regarding sex. In addition, although exposures were repeated, the study examined only a single dose of ethanol and yohimbine (4.0 g/kg and 2.0 mg/kg, respectively), which limits conclusions regarding potential dose‐dependent effects. The behavioral battery was restricted to two approach‐avoidance paradigms (LDB and EPM), so additional tasks probing other aspects of decision‐making, impulsivity, or threat evaluation may help determine the generality of the observed behavioral reorganization. Finally, yohimbine represents a pharmacological stressor and may not fully capture the complexity of naturalistic stress experiences.

In summary, our findings demonstrate that repeated ethanol intoxication or stress exposure during late adolescence leads to persistent impairments in risk assessment behavior, characterized by reduced cautious evaluation and altered behavioral sequencing in early adulthood. Importantly, these alterations emerged despite relatively limited changes in classical measures of anxiety‐like behavior, revealing a dissociation between traditional behavioral metrics and the finer‐grained organization of behavioral sequences captured by Markov chain analyses. These results highlight late adolescence as a critical window during which alcohol and stress can bias decision‐making strategies and reshape the structure of defensive behavior, with potential implications for understanding the long‐term behavioral consequences of early‐life exposures.

## Funding

This work was supported by Fundação de Amparo à Pesquisa do Estado de São Paulo (FAPESP grant #2019/01686‐0) and Coordenação de Aperfeiçoamento de Pessoal de Nível Superior (CAPES, student grant #88887.695072/2022‐00).

## Conflicts of Interest

The authors declare no conflicts of interest.

## Supporting information


**Table S1:** List of analyzed behaviors during the LDB and EPM tests.
**Table S2:** Significant differences in Markov chains' 1^st^ order transition probabilities for the Light–Dark Box test. (A) GzLM analysis comparing Ctrl and EtOH. (B) GzLM analysis comparing Ctrl and Yoh.
**Table S3:** Significant differences in Markov chains' 1^st^ order transition probabilities for the Elevated Plus Maze test. (A) GzLM analysis comparing Ctrl and EtOH. (B) GzLM analysis comparing Ctrl and Yoh.
**Figure S1:** Exploratory behaviors of adult female and male mice (9th weeks old) in the light side of the LDB 5 days after saline (Ctrl), ethanol (EtOH), or yohimbine (Yoh) exposures during late adolescence. (A) The analysis of the number of walks showed a significant effect of sex, as females engaged in more walks than males (X^2^_(1)_ = 4.07, *p* = 0.044). Sex‐separated analyses showed no significant differences among groups in females (X^2^_(2)_ = 0.83), but males from the EtOH group engaged in less walks than the Yoh group (X^2^_(2)_ = 8.80, *p* = 0.012; EtOH vs. Yoh: *p* = 0.011). (B) There was no significant effect of sex on the number of sniffs (X^2^_(1)_ = 1.99), and also no group effects following sex‐separated analyses (females: X^2^_(2)_ = 0.23; males: X^2^_(2)_ = 4.44). (C) Rears were increased in females compared to males (X^2^_(1)_ = 9.31, *p* = 0.002). Sex‐separated analyses showed that the number of rears was not affected by treatment in females, whereas males in the EtOH group did less rears than Ctrl and Yoh. * *p* < 0.05. Ctrl: *n* = 10/sex; EtOH: *n* = 10/sex; Yoh: *n* = 10 females, 9 males. Data are presented as mean ± SEM.
**Figure S2:** Exploratory behaviors of adult female and male mice (9 weeks old) in the EPM 7 days after saline (Ctrl), ethanol (EtOH) or yohimbine (Yoh) exposures during late adolescence. (A) Walks in the closed arms were similar in both sexes (X^2^_(1)_ = 0.05). Sex‐separated analyses showed no group effects (females: X^2^_(2)_ = 2.01; males: X^2^_(2)_ = 0.33). (B) Females walked more in the open arms in comparison to males (X^2^_(1)_ = 5.31, *p* = 0.021), regardless of group. Walks in the open were not affected by treatment in females (X^2^_(2)_ = 0.33), whereas EtOH males performed fewer walks in the open arm in comparison to Ctrls (X^2^_(2)_ = 7.91, *p* = 0.019). (C) Sniffs in the closed arms did not change across sexes (X^2^_(1)_ = 2.61), and sex‐separated analyses showed no group effects (females: X^2^_(2)_ = 2.40; males: X^2^_(2)_ = 3.01). (D) Sniffs in the open arms did not change across sexes (X^2^_(1)_ = 0.03), and sex‐separated analyses showed no group effects (females: X^2^_(2)_ = 3.22; males: X^2^_(2)_ = 0.33). (E) Females did more rears than males (X^2^_(1)_ = 4.35, *p* = 0.037), regardless of group. Sex‐separated analyses showed no group effects (females: X^2^_(2)_ = 1.74; males: X^2^_(2)_ = 0.68). (F) Head dips were similar in both sexes (X^2^_(1)_ = 1.18). Sex‐separated analyses showed no group effects (females: X^2^_(2)_ = 0.29; males: X^2^_(2)_ = 2.75). Ctrl: *n* = 10/sex; EtOH: *n* = 10 females, 9 males; Yoh: *n* = 10 females, 9 males. Data are presented as mean ± SEM.
**Figure S3:** Distance and velocity during the EPM test following saline (Ctrl), ethanol (EtOH) and yohimbine (Yoh) exposures during late adolescence and adulthood. (A) Total distance traveled did not differ across groups of mice exposed during late adolescence (X^2^_(2)_ = 0.64). (B) Velocity was also similar in all groups (X^2^_(2)_ = 0.53). Data are presented as mean ± SEM.

## Data Availability

Supporting data are available from the corresponding author on reasonable request.
