# Supplementary material for: Alcohol or Stress Exposure During Late Adolescence Impairs Risk Assessment Later in Life, Disrupting the Ethological Structure of Behavior in Mice
Source: Alcohol Clin Exp Res (Hoboken). 2026 Jul 10;50(7):e70328. doi: 10.1111/acer.70328 (PMC13353261; doi:10.1111/acer.70328)
Supplement: Supplementary file 1 — Table S1: List of analyzed behaviors during the LDB and EPM tests. Table S2: Significant differences in Markov chains' 1st order transition probabilities for the Light–Dark Box test. (A) GzLM analysis comparing Ctrl and EtOH. (B) GzLM analysis comparing Ctrl and Yoh. Table S3: Significant differences in Markov chains' 1st order transition probabilities for the Elevated Plus Maze test. (A) GzLM analysis comparing Ctrl and EtOH. (B) GzLM analysis comparing Ctrl and Yoh. Figure S1: Exploratory behaviors of adult female and male mice (9th weeks old) in the light side of the LDB 5 days after saline (Ctrl), ethanol (EtOH), or yohimbine (Yoh) exposures during late adolescence. (A) The analysis of the number of walks showed a significant effect of sex, as females engaged in more walks than males (X2 (1) = 4.07, p = 0.044). Sex‐separated analyses showed no significant differences among groups in females (X2 (2) = 0.83), but males from the EtOH group engaged in less walks than the Yoh group (X2 (2) = 8.80, p = 0.012; EtOH vs. Yoh: p = 0.011). (B) There was no significant effect of sex on the number of sniffs (X2 (1) = 1.99), and also no group effects following sex‐separated analyses (females: X2 (2) = 0.23; males: X2 (2) = 4.44). (C) Rears were increased in females compared to males (X2 (1) = 9.31, p = 0.002). Sex‐separated analyses showed that the number of rears was not affected by treatment in females, whereas males in the EtOH group did less rears than Ctrl and Yoh. * p < 0.05. Ctrl: n = 10/sex; EtOH: n = 10/sex; Yoh: n = 10 females, 9 males. Data are presented as mean ± SEM. Figure S2: Exploratory behaviors of adult female and male mice (9 weeks old) in the EPM 7 days after saline (Ctrl), ethanol (EtOH) or yohimbine (Yoh) exposures during late adolescence. (A) Walks in the closed arms were similar in both sexes (X2 (1) = 0.05). Sex‐separated analyses showed no group effects (females: X2 (2) = 2.01; males: X2 (2) = 0.33). (B) Females walked more in the open arms in compar [file ACER-50-0-s001.pdf]

Supplemental information

Alcohol or stress exposure during late adolescence impairs risk assessment later in life, disrupting the ethological structure of behavior in mice

Beatriz Deo Sorigotto[1], MSc; Isabel Marian Hartmann de Quadros[1,2], PhD; Karina Possa Abrahao[1,3]*, PhD

[1] Programa de Pós-Graduação em Psicobiologia, Departamento de Psicobiologia, Escola Paulista de Medicina, Universidade Federal de São Paulo, São Paulo, São Paulo, Brasil.

[2] Departamento de Psicobiologia, Escola Paulista de Medicina, Universidade Federal de São Paulo, São Paulo, São Paulo, Brasil.

[3] Departamento de Fisiologia, Escola Paulista de Medicina, Universidade Federal de São Paulo, São Paulo, São Paulo, Brasil.

* Correspondent author

Table S1: List of analyzed behaviors during the LDB and EPM tests.

BEHAVIOR	CATEGORY	DEFINITION
Rear	Exploratory	The mouse assumes a vertical position supporting itself on its hind legs while the front legs either move in the air or lean against a wall.
Head dip	Exploratory	Downward visual screening that happens when the mouse is in one of the elevated open arms of the plus maze.
Walk	Exploratory	The mouse moves around and changes position. Includes non-specific body movements such as turning or running across the apparatus.
Sniff	Exploratory	Rhythmic inhalation and exhalation of air through the nose while the mouse stands still.
Grooming*	Self-care	The mouse rubs any part of the face with circular movements of the forepaws, licks its body and/or its forepaws and back paws.
Light side entry	Exploratory	The mouse enters the light side of the LDB with its four paws.
Dark side entry	Exploratory	The mouse enters the dark side of the LDB with its four paws.
Open arm entry	Exploratory	The mouse moves from the central platform to an open arm.
Closed arm entry	Exploratory	The mouse moves from the central platform to a closed arm.
NoGo Head-out **	Risk assessment	While in a protected area, the mouse extends only its head towards the aversive area, then returns to the original position.
Go Head-out**	Risk assessment	While in a protected area, the mouse extends only its head toward the aversive zone before fully entering it.
NoGo Stretch**	Risk assessment	Forward extension of the head and one or both forepaws from a protected area towards an aversive area followed by retraction to the original position.
Go Stretch**	Risk assessment	Forward extension of the head and one or both forepaws from a protected area towards an aversive zone before fully entering it.

*Occurred less than 1% of the time during the LDB and EPM tests.
**In the EPM, head-out and stretch postures occur in the closed arms or central platform in direction to the open arm, while in the LDB these behaviors occur when the animal is in the dark compartment and extends its body towards the light side.

Table S2: Significant differences in Markov chains' first order transition probabilities for the Light-Dark Box test. **(A)** GzLM analysis comparing Ctrl and EtOH. **(B)** GzLM analysis comparing Ctrl and Yoh.

A. EtOH vs. Ctrl

Transition		Prob. mean	SD	$\chi^2_{(1)}$	p
Dark entry - HO NoGo	Ctrl	0.033	0.015	9.63	0.002
	EtOH	0.020	0.012		
Dark entry - HO Go	Ctrl	0.011	0.012	9.76	0.002
	EtOH	0.027	0.017		
Dark entry - ST Go	Ctrl	0.014	0.015	8.12	0.004
	EtOH	0.030	0.021		
HO NoGo - HO NoGo	Ctrl	0.008	0.010	4.54	0.033
	EtOH	0.002	0.004		
Rearing - walking	Ctrl	0.138	0.036	4.12	0.042
	EtOH	0.120	0.025		
ST NoGo - ST NoGo	Ctrl	0.009	0.013	5.84	0.016
	EtOH	0.001	0.004		

B. Yoh vs. Ctrl

Transition		Prob. mean	SD	$\chi^2_{(1)}$	p
Dark entry - HO NoGo	Ctrl	0.033	0.015	7.94	0.005
	Yoh	0.019	0.015		
Dark entry - ST NoGo	Ctrl	0.024	0.015	4.57	0.032
	Yoh	0.015	0.012		
Dark entry - ST Go	Ctrl	0.014	0.015	6.56	0.010
	Yoh	0.027	0.018		
Sniffing - walking	Ctrl	0.034	0.017	5.6	0.018
	Yoh	0.049	0.022		
ST NoGo - HO NoGo	Ctrl	0.010	0.015	6.55	0.010
	Yoh	0.001	0.004		
ST NoGo - ST NoGo	Ctrl	0.009	0.013	5.47	0.019
	Yoh	0.002	0.005		

HO: head-out; ST: stretch; Ctrl: control; EtOH: ethanol; Yoh: yohimbine. Ctrl: n = 10/sex; EtOH: n = 10/sex; Yoh: n = 10 females, 9 males.

Table S3: Significant differences in Markov chains' first order transition probabilities for the Elevated Plus Maze test. **(A)** GzLM analysis comparing Ctrl and EtOH. **(B)** GzLM analysis comparing Ctrl and Yoh.

A. <u>EtOH vs. Ctrl</u>

Transition		Prob. mean	SD	$\chi^2_{(1)}$	p
HO NoGo - walking in closed arm	Ctrl	0.018	0.014	4.93	0.026
	EtOH	0.007	0.012		
Sniffing in closed arm - rearing	Ctrl	0.045	0.018	5.73	0.017
	EtOH	0.033	0.013		
ST NoGo - ST NoGo	Ctrl	0.007	0.009	5.70	0.017
	EtOH	0.002	0.004		
Walking in closed arm - HO NoGo	Ctrl	0.027	0.013	5.80	0.016
	EtOH	0.015	0.014		

B. <u>Yoh vs. Ctrl</u>

Transition		Prob. mean	SD	$\chi^2_{(1)}$	p
Sniffing in closed arm - rearing	Ctrl	0.045	0.018	4.23	0.040
	Yoh	0.033	0.014		
ST NoGo - HO NoGo	Ctrl	0.003	0.004	6.43	0.011
	Yoh	0.000	0.000		
Walking in closed arm - HO NoGo	Ctrl	0.027	0.013	10.36	0.001
	Yoh	0.002	0.014		

HO: head-out; ST: stretch; Ctrl: control; EtOH: ethanol; Yoh: yohimbine. Ctrl: n = 10/sex; EtOH: n = 10 females, 9 males; Yoh: n = 10 females, 9 males.

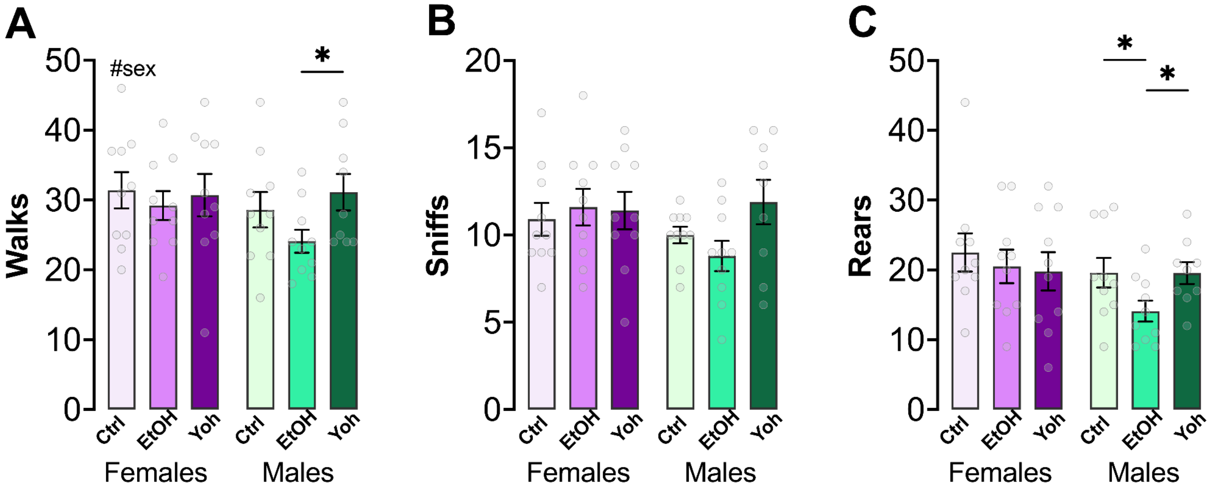

Figure S1. Exploratory behaviors of adult female and male mice (9th weeks old) in the light side of the LDB five days after saline (Ctrl), ethanol (EtOH) or yohimbine (Yoh) exposures during late adolescence. (A) The analysis of the number of walks showed a significant effect of sex, as females engaged in more walks than males ($X^2_{(1)} = 4.07$, $p = 0.044$). Sex-separated analyses showed no significant differences among groups in females ($X^2_{(2)} = 0.83$), but males from the EtOH group engaged in less walks than the Yoh group ($X^2_{(2)} = 8.80$, $p = 0.012$; EtOH vs. Yoh: $p = 0.011$). **(B)** There was no significant effect of sex on the number of sniffs ($X^2_{(1)} = 1.99$), and also no group effects following sex-separated analyses (females: $X^2_{(2)} = 0.23$; males: $X^2_{(2)} = 4.44$). **(C)** Rears were increased in females compared to males ($X^2_{(1)} = 9.31$, $p = 0.002$). Sex-separated analyses showed that the number of rears was not affected by treatment in females, whereas males in the EtOH group did less rears than Ctrl and Yoh. * $p < 0.05$. Ctrl: n = 10/sex; EtOH: n = 10/sex; Yoh: n = 10 females, 9 males. Data are presented as mean ± SEM.

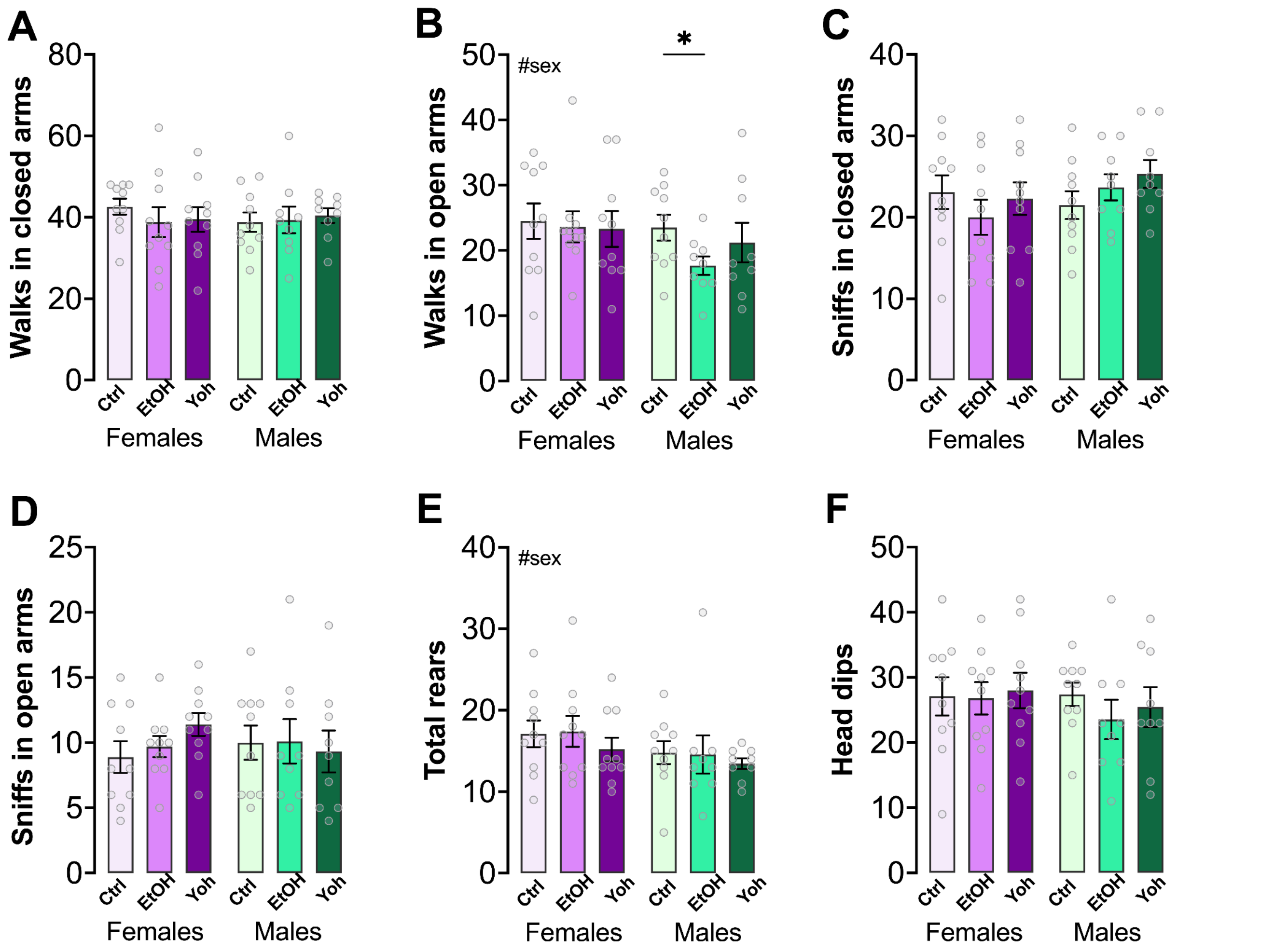

Figure S2. Exploratory behaviors of adult female and male mice (9 weeks old) in the EPM seven days after saline (Ctrl), ethanol (EtOH) or yohimbine (Yoh) exposures during late adolescence. **(A)** Walks in the closed arms were similar in both sexes ($X^2_{(1)}$ = 0.05). Sex-separated analyses showed no group effects (females: $X^2_{(2)}$ = 2.01; males: $X^2_{(2)}$ = 0.33). **(B)** Females walked more in the open arms in comparison to males ($X^2_{(1)}$ = 5.31, p = 0.021), regardless of group. Walks in the open were not affected by treatment in females ($X^2_{(2)}$ = 0.33), whereas EtOH males performed fewer walks in the open arm in comparison to Ctrls ($X^2_{(2)}$ = 7.91, p = 0.019). **(C)** Sniffs in the closed arms did not change across sexes ($X^2_{(1)}$ = 2.61), and sex-separated analyses showed no group effects (females: $X^2_{(2)}$ = 2.40; males: $X^2_{(2)}$ = 3.01). **(D)** Sniffs in the open arms did not change across sexes ($X^2_{(1)}$ = 0.03), and sex-separated analyses showed no group effects (females: $X^2_{(2)}$ = 3.22; males: $X^2_{(2)}$ = 0.33). **(E)** Females did more rears than males ($X^2_{(1)}$ = 4.35, p = 0.037), regardless of group. Sex-separated analyses showed no group effects (females: $X^2_{(2)}$ = 1.74; males: $X^2_{(2)}$ = 0.68). **(F)** Head dips were similar in both sexes ($X^2_{(1)}$ = 1.18). Sex-separated analyses showed no group effects (females: $X^2_{(2)}$ = 0.29; males: $X^2_{(2)}$ = 2.75). Ctrl: n = 10/sex; EtOH: n = 10 females, 9 males; Yoh: n = 10 females, 9 males. Data are presented as mean ± SEM.

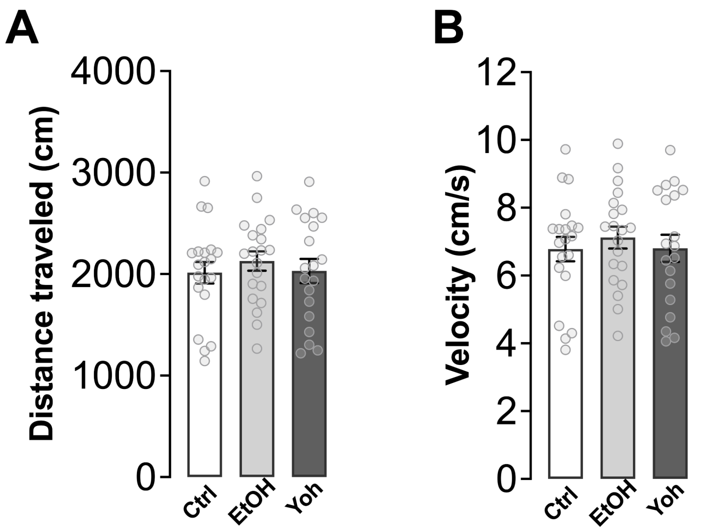

Figure S3. Distance and velocity during the EPM test following saline (Ctrl), ethanol (EtOH) and yohimbine (Yoh) exposures during late adolescence and adulthood. (A) Total distance traveled did not differ across groups of mice exposed during late adolescence ($X^2_{(2)}$ = 0.64). **(B)** Velocity was also similar in all groups ($X^2_{(2)}$ = 0.53). Data are presented as mean ± SEM.
